# A KNOWLEDGE GRAPH–BASED DEMENTIA CARE INTELLIGENT RECOMMENDER SYSTEM FOR MANAGING DEMENTIA CARE

**DOI:** 10.1093/geroni/igae098.3544

**Published:** 2024-12-31

**Authors:** Yue Sun, Zhi-wen Wang

**Affiliations:** Peking University, Beijing, Beijing, China (People’s Republic); Peking University Health Science Center, Beijing, Beijing, China (People’s Republic)

## Abstract

**Background:**

Caring for people with dementia is perceived as one of the most challenging caring roles, so effective and practical support is essential. One such innovative approach to internet-based tailored health intervention is the use of recommender system. Objective: This study develops a mobile-based dementia care intelligent recommender system (DCIRS) that can provide personalized and timely care interventions recommendations for caregivers when they encounter difficult various care problems.

**Methods:**

A knowledge graph was constructed using the 1023 personalized care plan set for dementia patients as the source material. The graph embedding method was used to introduce the constructed domain knowledge graph of dementia care into the recommender model. The graph embedding module learned the features of the knowledge graph through the TransR translation model, and the recommender module interacts with the learned features of the graph and the recommended items to realize personalized recommendation of care plans with the help of the above recommender algorithm.

**Results:**

The core function of the DCIRS was to recommend personalized care plans according to the personalized characteristics of people with dementia, such as gender, disease severity, hobbies, walking ability, and care problems. Conclusions: This study was a pioneering research to develop a comprehensive recommender system for caregivers of people with dementia. According to the evaluation results, our DCIRS showing high specificity and accuracy. This system can provide a novel perspective for patient-centered and needs-based support of caregivers of people with dementia.

